# Diagnosis of Glaucoma Based on Few-Shot Learning with Wide-Field Optical Coherence Tomography Angiography

**DOI:** 10.3390/biomedicines12040741

**Published:** 2024-03-27

**Authors:** Kyoung Ok Yang, Jung Min Lee, Younji Shin, In Young Yoon, Jun Won Choi, Won June Lee

**Affiliations:** 1 Department of Artificial Intelligence, Hanyang University, Seoul 04763, Republic of Korea; koyang@spa.hanyang.ac.kr; 2 Department of Ophthalmology, Hanyang University Seoul Hospital, Seoul 04763, Republic of Korea; jjungmin250@gmail.com; 3 Department of Electrical Engineering, Hanyang University, Seoul 04763, Republic of Korea; yjshin@spa.hanyang.ac.kr (Y.S.); inyoungyoon@spa.hanyang.ac.kr (I.Y.Y.); 4 Department of Electrical and Computer Engineering, College of Liberal Studies, Seoul National University, Seoul 08826, Republic of Korea; 5 Department of Ophthalmology, Hanyang University College of Medicine, Seoul 04763, Republic of Korea

**Keywords:** deep learning, image processing, glaucoma, diagnostic ability, few-shot learning

## Abstract

This study evaluated the utility of incorporating deep learning into the relatively novel imaging technique of wide-field optical coherence tomography angiography (WF-OCTA) for glaucoma diagnosis. To overcome the challenge of limited data associated with this emerging imaging, the application of few-shot learning (FSL) was explored, and the advantages observed during its implementation were examined. A total of 195 eyes, comprising 82 normal controls and 113 patients with glaucoma, were examined in this study. The system was trained using FSL instead of traditional supervised learning. Model training can be presented in two distinct ways. Glaucoma feature detection was performed using ResNet18 as a feature extractor. To implement FSL, the ProtoNet algorithm was utilized to perform task-independent classification. Using this trained model, the performance of WF-OCTA through the FSL technique was evaluated. We trained the WF-OCTA validation method with 10 normal and 10 glaucoma images and subsequently examined the glaucoma detection effectiveness. FSL using the WF-OCTA image achieved an area under the receiver operating characteristic curve (AUC) of 0.93 (95% confidence interval (CI): 0.912–0.954) and an accuracy of 81%. In contrast, supervised learning using WF-OCTA images produced worse results than FSL, with an AUC of 0.80 (95% CI: 0.778–0.823) and an accuracy of 50% (*p*-values < 0.05). Furthermore, the FSL method using WF-OCTA images demonstrated improvement over the conventional OCT parameter-based results (all *p*-values < 0.05). This study demonstrated the effectiveness of applying deep learning to WF-OCTA for glaucoma diagnosis, highlighting the potential of WF-OCTA images in glaucoma diagnostics. Additionally, it showed that FSL could overcome the limitations associated with a small dataset and is expected to be applicable in various clinical settings.

## 1. Introduction

Glaucoma refers to a disease that involves specific morphologic changes in the optic nerve resulting in functional changes in the visual field due to loss of the retinal nerve fiber layer (RNFL) [1,2]. Disc photography, optical coherence tomography (OCT) [3,4,5,6,7,8,9], and OCT angiography (OCTA) are among the various imaging devices used for diagnosing glaucoma. The diagnostic data are presented as images or numerical values, depending on the instrument used. Among these techniques, OCTA is a non-invasive imaging method that assesses the vasculature of the retina and optic nerve without the need for dye injection [10,11]. Changes in vessel density in OCTA align with functional and structural alterations detected through visual field exams and OCT scans, providing good consistency and effectively distinguishing between the glaucomatous and the normal eyes.

Wide-field OCTA (WF-OCTA), which overcomes the limited field of view in traditional OCTA, is emerging as one of the new diagnostic imaging approaches for retinal disease and glaucoma [12,13,14,15,16]. WF-OCTA’s scanning capabilities have been improved with technical advancements, such as swept-source OCT (SS-OCT), now allowing the examination of large areas of the posterior pole, encompassing both the optic nerve head and macula. Notably, when examining pathologic eyes with structural distortion of the optic disc, such as high myopia or retinal diseases, including epiretinal membrane and peripapillary retinoschisis, errors may occur in measuring conventional RNFL thickness maps. Additionally, WF-OCTA displays broader angiographic data in comparison to conventional imaging. This could potentially enhance the accuracy of glaucoma diagnosis, especially when other pathological alterations in the eyes complicate the process.

This study evaluates the accuracy of a deep-learning (DL) algorithm using WF-OCTA for identifying glaucoma. DL image classification is being assessed as a pre-diagnostic tool before human diagnosis. Sufficient data are crucial for effectively training DL networks for image classification in medical imaging diagnosis. Insufficient data can result in issues like overfitting and underfitting. Collecting sufficient medical data for training is a challenge due to limited data availability and privacy concerns. Furthermore, the clinical stage of WF-OCTA—the technology utilized in this study—creates difficulties in obtaining adequate data.

In recent years, few-shot learning (FSL) has emerged as a promising approach in DL, particularly in scenarios where limited annotated data are available. Unlike traditional supervised learning methods, which rely on large, labeled datasets for training, FSL enables models to generalize to new tasks with only a small amount of annotated data, mimicking human learning processes with limited examples [17]. The relationship between dataset size and accuracy in machine learning, including FSL, is complex. While larger datasets typically offer more diverse examples for model training, several factors impact this relationship. High-quality, well-annotated data are crucial for training accurate models, and task complexity and model architecture also influence performance [18]. Imbalanced data distributions and the use of regularization techniques further shape the interplay between dataset size and accuracy [19]. In the context of FSL, dataset size plays a crucial role in model performance. Although FSL techniques can handle limited data scenarios, increasing the dataset size can significantly enhance performance, especially if the additional data includes rare cases or provides greater diversity [20]. It is essential to understand these dynamics to optimize model performance and effectively utilize available data resources.

In such situations, implementing the FSL [21,22,23] approach may be a way to overcome this challenge. FSL methodology permits machine learning from a small number of samples, usually less than 10. Therefore, this study assessed the diagnostic potential of WF-OCTA for detecting glaucoma using an FSL approach to overcome data scarcity.

## 2. Materials and Methods

This study’s protocol was approved by the Institutional Review Board (IRB) of Hanyang University Hospital, Seoul, Republic of Korea (IRB number: HYUH 2021-07-036). This study was designed in accordance with the tenets of the Declaration of Helsinki for biomedical research. The need for participant consent for retrospective data assessment was waived by the ethics committee.

### 2.1. Study Design and Participants

In this retrospective, comparative study, a total of 195 eyes were examined at Hanyang University Seoul Hospital Glaucoma Clinic between December 2021 and December 2022. Of these, 82 eyes were affected with glaucoma, and 113 controls were without glaucoma. All participants underwent WF-OCTA imaging with the same SS-OCT device (Topcon, DRI OCT Triton), and the glaucoma was diagnosed by a glaucoma specialist. Diagnosis of glaucoma and selection of the control group were performed similarly to that in previous studies (Supplementary Materials) [24,25]. To eliminate ambiguity, this study excluded patients with high myopia (sph < −6.0D), retinal diseases, and glaucoma suspect states without definite visual field impairment or RNFL defects.

### 2.2. WF-OCTA

The wide-field 12 × 12 mm OCTA scan generates an en-face image of retinal vessels through various segmented layers. The SS-WF-OCTA scans volumes centered on the retina within a 12 × 12 mm field of view at a scan rate of 100,000 A-scans per second, offering a lateral resolution of 20 um. The device’s built-in software corrects actual refraction to prevent refractometric degradation. The report of the WF-OCTA scan for the 12 × 12 area overlaps with the RNFL or ganglion cell–inner plexiform layer (GCIPL)/ganglion cell complex (GCC) thickness map on the WF-OCTA image.

**Figure 1:**
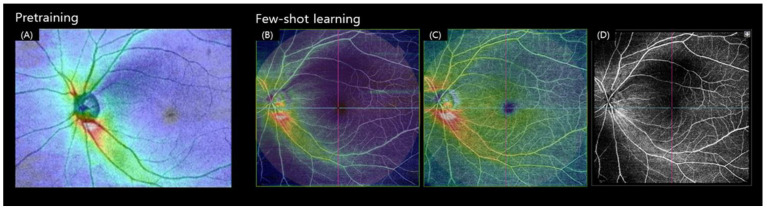
Dataset examples for pre-training and FSL: (**A**) SS-OCT RNFL thickness map (12 × 9 mm) used in pretraining, (**B**) WF-OCTA-RNFL Combi, (**C**) WF-OCTA-GCC Combi, and (**D**) WF-OCTA used for FSL.

Figure 1 displays (A) the OCT RNFL thickness map used in pre-training and the three types of WF-OCTA images in the FSL, (B) a combination of WF-OCTA and RNFL thickness map (Combi 1), (C) a combination of WF-OCTA and GCC thickness map (Combi 2), and (D) WF-OCTA itself (black and white). Apart from the WF-OCTA, this work evaluated the vessel density using optic disc OCTA (4.5 × 4.5 mm). The vessel density of the superficial capillary plexus (SCP) was assessed in four groups, superior, nasal, inferior, and temporal, to determine whether the vessel density had decreased.

### 2.3. DL Techniques: Image Classification on Medical Diagnosis

Medical image classification is pivotal in clinical treatment and early diagnosis. However, traditional methods have demonstrated limited performance and often require significant time and effort to identify and select features for classification. DL methods have surpassed the performance of some existing models and streamlined the design process through a data-driven approach. In particular, the supervised learning (SL) method using DL networks has achieved great success in various image classification tasks [26,27,28]. As the parameters of DL networks for image classification tend to be large, a large amount of training data is required to train networks. However, when it comes to medical image classification, the preparation of extensive training datasets demands costly and time-consuming manual annotations by medical professionals. Moreover, the distribution of medical data can be significantly imbalanced. Acquiring a vast amount of normal data might be easy; however, obtaining negative samples is challenging due to the rarity of certain disease cases. To address this issue, a training approach capable of accurately diagnosing diseases with a very limited amount of data was required. FSL aims to make predictions in situations where only a few examples are available for each class [29]. This study presents an effective glaucoma detection method leveraging the FSL method.

**Figure 2:**
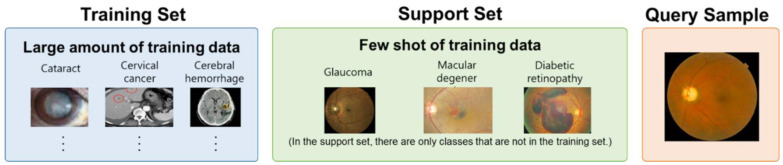
Categorization of datasets in FSL. The training set is used to train a deep-learning network to extract task-specific features from images. The support set is a small set of data points (e.g., 1, 2, 5, 10, etc.) used for training an FSL model for a new task. The query sample is data to evaluate the effectiveness of a network trained on a limited dataset.

Data used in FSL can be broadly classified into three categories: training set, support set, and query set [30] (Figure 2). Additionally, the ‘class’ used in FSL refers to the group of objects that the model is trying to learn and distinguish from other classes. FSL initially learns how to distinguish between classes through the training set with a large amount of data. Then, through the support set, FSL learns from only a small amount of data for each class that is not present in the training set. When a query sample is input, the system compares which class of the support set is most similar to the answer.

ProtoNet [31] is an FSL method that uses a prototype that represents each class or concept as a central reference point in the feature space. During training, the model learns to create a prototype from a few labeled examples, using DL networks such as convolutional neural networks to extract meaningful features from the input data. The prototype is computed as the average feature vector of the supporting examples belonging to each class, and during the inference step, the model compares the query example to the prototype and assigns it to the class with the closest prototype. ProtoNet utilizes a metric learning objective function, enabling effective learning with limited data. This capability allows the model to differentiate between various classes and apply to new instances.

### 2.4. DL Techniques: Proposed Method

This study proposes a ProtoNet-based network for predicting WF-OCTA through DL. The ProtoNet-based network comprises a feature-extracting backbone network and a true/false classifying clustering module. The backbone network varies based on the size and type of data in both networks. Moreover, ResNet18 was deployed as our backbone network [27].

ResNet18 is trained on a very large dataset, such as ImageNet [32] (1.2 million images stored in 1000 categories), using a pre-trained model [33]. We can utilize the pre-trained model as an initialization or a fixed feature extractor and employ the transfer learning method [34]. Transfer learning is a DL method in which a model trained for one task is repurposed for a related second task. We utilized the weight of the backbone network by applying this fine-tuning of the transfer learning method with SS-OCT images [24,25] using ResNet18 pre-trained with ImageNet (Figure S1). For pre-training, the SS-OCT RNFL thickness map (12 × 9 mm) of glaucoma and normal groups was used (Figure 1). The data from the patient group in our existing study (*Journal of Glaucoma*) was used [25], and a total of 417 eyes with glaucoma and 258 normal eyes were included.

From this point forward, this discussion focuses on the process of acquiring knowledge about ProtoNet’s clustering module through training and testing. To train the clustering module, Mini-ImageNet [35], a modified version of ImageNet for FSL, was utilized. Training with WF-OCTA introduces a potential risk of overfitting, as the model may become too specialized in WF-OCTA data. As each patient demonstrates different glaucoma symptoms in the data, overfitting the training data increases the risk of incorrect predictions for new patients. To mitigate this, we trained ProtoNet on Mini-ImageNet to establish a general understanding of the difference between similarity and dissimilarity.

**Figure 3:**
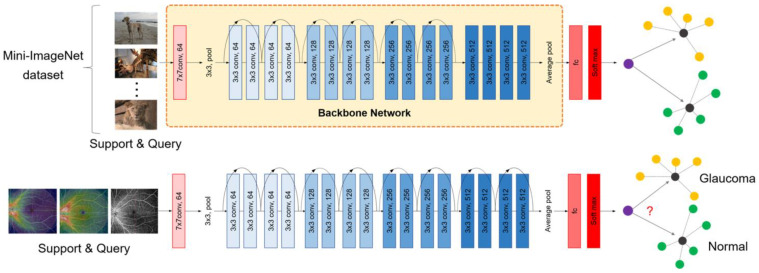
Implementation of FSL for WF-OCTA integration. This study employed FSL to integrate WF-OCTA into an AI algorithm. ResNet18 served as the backbone network, trained on ophthalmic images to capture relevant features. The ProtoNet algorithm facilitated feature clustering and classification. Training utilized the Mini-ImageNet benchmark dataset. The WF-OCTA data were split into support and query sets for validation.

Finally, the network’s performance on WF-OCTA data was evaluated in the test or inference phase. We trained the neural network on WF-OCTA data from 10 patients with glaucoma and 10 normal individuals, forming a support set. Few-shot training, in this case, refers to the adaptation of a pre-existing network to a new task with minimal input, specifically for glaucoma. Subsequently, we supplemented the set with 100 unseen data points, referred to as the query set, and evaluated whether each data point represented glaucoma or not. The schematic diagram is presented in Figure 3.

### 2.5. Statistical Analysis

This study compared the diagnostic performance between FSL conducted with pre-training on different images and Mini-ImageNet and conventional SL using a limited number of WF-OCTA images. Furthermore, this study extended the comparison to evaluate the diagnostic capabilities of numerical values (parameters) commonly used in traditional OCT and OCTA.

To assess the diagnostic capability for detecting the presence or absence of glaucoma, we computed the area under the receiver operating characteristic curve (AUC) and accuracy. Additionally, AUC with a 95% confidence interval (95% CI) was employed while varying the cutoff value for the probability of glaucoma. The method described by DeLong et al. [36] was utilized to compare AUC values among different parameters. Accuracy served as a metric for the precision in classifying the stages of glaucoma. The proportion of correctly classified data from the entire dataset used for testing was also estimated. *p*-values < 0.05 were considered statistically significant. Values are presented as mean ± standard deviation. Statistical tests were conducted using SPSS version 24 (IBM Inc., Armonk, NY, USA), MedCalc Version 19.1.3 (MedCalc Software, Ostend, Belgium), and the PyTorch Version 1.12.0 in Python (Facebook AI Research Lab, Menlo Park, CA, USA) [37].

## 3. Results

Demographics and ocular characteristics of the support set and query set are summarized in Table S1. The median age was 58.4 ± 15.8. No statistically significant differences were observed in spherical equivalent, axial length, and intraocular pressure, regardless of the presence of glaucoma. Both glaucoma and control groups were evenly composed with similar numerical values of other ocular characteristics such as MD (dB), VFI (%), RNFL, GCIPL, and GCC thickness (μm).

In the experiment, 20 WF-OCTA images, consisting of 10 glaucoma and 10 normal datasets, were examined. Additionally, 100 WF-OCTA images of 50 glaucoma and 50 normal datasets were classified in FSL experiments with 1, 2, 5, and 10 shots by default. Shot refers to the number of data points used to adapt training to a new task. For example, if it is one shot, it means that the model only looks at one glaucoma data and one normal data and fits 100 data. Table S2 demonstrates the WF-OCTA image accuracy value and the AUC. The results clearly display that as the number of shots increases, so does the accuracy. The comparison was based on 10 shots.

**Figure 4:**
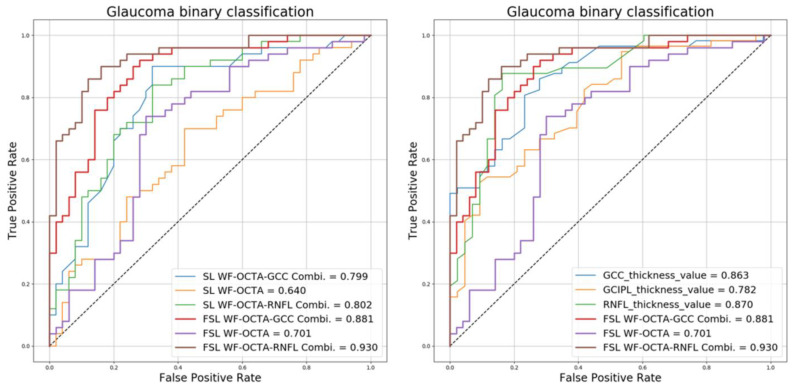
Receiver operating curves for glaucoma diagnostic methods. Left is FSL vs. SL with WF-OCTA images. FSL shows a higher AUC. The right is a classification of glaucoma vs. normal cases. FSL WF-OCTA RNFL Combi and FSL WF-OCTA GCC Combi achieve the highest AUC (0.930 and 0.881). These outperform conventional thickness values (RNFL, GCC, and GCIPL thickness AUC: 0.870, 0.863, and 0.782).

We were interested in the feasibility of using SL to predict WF-OCTA even with a limited amount of data. To investigate this point, we conducted a performance verification and comparison between the existing SL method using ResNet18 and the proposed method with the WF-OCTA data. For SL, we trained with a total of 20 WF-OCTA data and tested with 100 WF-OCTA data. Therefore, Figure 4 and Table 1 demonstrate that SL does not learn with an accuracy of 50%. Additionally, the accuracy and the AUC value are high for FSL, and the *p*-value is set at 0.05 for all results, which is significant.

This study also compared our method with existing methods based on peripapillary RNFL, macular GCIPL, and macular GCC thickness values, which are widely used in the glaucoma field.

As displayed in Table 2, the AUC values demonstrate that the performance of WF-OCTA Combis adopting FSL is significantly higher than those of thickness values, and the respective *p*-values are significant at 0.05 or less.

## 4. Discussion

In this study, the effectiveness of WF-OCTA as an image diagnostic modality for glaucoma diagnosis is investigated using DL algorithms, and an FSL method is also introduced to overcome the difficulties of data collection for WF-OCTA. Although many previous studies have applied artificial intelligence (AI) to images such as OCT and SS-OCT to diagnose glaucoma, no studies have been conducted on WF-OCTA images. Therefore, the application of DL to WF-OCTA images is the first of its kind.

In the field of ophthalmology, various attempts have been made to use small-shot learning as an automatic diagnostic evaluation method for images. Quellec et al. applied FSL to the detection of rare conditions such as papilledema or anterior ischemic optic neuropathy from the OPHDIAT diabetic retinopathy screening program [38]. Kim et al. introduced a novel approach for the development of an effective computational model for early diagnosis of glaucoma, relying solely on a single type of image (high-resolution fundus images) using FSL, employing a matching network older than ProtoNet [39]. Han et al. used FSL for ophthalmic disease screening, focusing primarily on enhancing data by fusion or aggregation of various data types rather than relying on a small amount of data [40].

First of all, comparing FSL and SL, the diagnostic power of FSL demonstrated better performance. The reason SL does not perform is because of an error called underfitting that occurs due to the small amount of image data (like WF-OCTA). This means that to examine the effectiveness of using AI in this situation, FSL, rather than the existing methodology SL, must be used as another methodology that can improve performance. This does not serve as a comparison to establish the superiority of SL over FSL; instead, it underscores the importance of FSL as a fitting methodology in specific circumstances. FSL was utilized to construct models by incorporating various data sources, including open benchmark data, to verify its effectiveness for WF-OCTA data.

This study conducted experiments on the FSL method utilizing an algorithm called ProtoNet, which transforms data into prototypes and clusters them, making them suitable for displaying particularity distributions. To adjust the ProtoNet for the FSL algorithm for this study, we identified two stages. The first step involves extracting features, which capture critical image elements to be evaluated, followed by classification, which determines whether results are positive or negative. The feature extractor was trained using RNFL and the transfer learning method [41], which utilized an existing ophthalmic medical image. This resulted in an effective feature extractor for ophthalmic medical images. To reduce potential task dependence, the model was trained on ImageNet during the classification process. Additionally, the use of distinct datasets for training both sections of the network aimed to maximize its benefits.

The FSL method employed in this study has the potential to significantly impact real-world medical applications. We think this is the result of improving FSL’s capabilities through the aforementioned efforts. FSL can be useful in cases where there are not enough images available for training due to the rarity of the disease or the release of new image types in the future. As new images will continue to be introduced with the development of technology, incorporating FSL will help research and evaluate the diagnostic power at the time of introduction of such images.

This study also compared the FSL of WF-OCTA with existing methods based on peripapillary RNFL, macular GCIPL, and macular GCC thickness values, which are widely used parameters in glaucoma. Application of WF-OCTA data to FSL, especially in WF-OCTA Combi data, demonstrated that this method surpasses conventional numeric parameter-based diagnosis. Nevertheless, WF-OCTA (not combi, alone, grayscale) exhibits decreased performance compared to the other two images. The reason for this discrepancy lies in the fact that the RNFL image used for feature training is in RGB format, while the other two images are in RGB format, and WF-OCTA is a grayscale image, thus degrading the feature extraction performance due to different channels. To enhance the efficiency of WF-OCTA (grayscale), acquire enough grayscale glaucoma images to train the feature extractor or use additional shots to increase the accuracy of the network. We expect that increasing the number of attempts beyond 10 may potentially enhance the algorithm’s performance in future research.

WF-OCTA offers several distinct advantages in detecting RNFL defects for glaucoma diagnosis [42]. First, WF-OCTA visualizes a wider area (12 × 12 mm) compared to the existing OCT RNFL thickness map (12 × 9 mm). This may also be useful in assessing peripheral regions and detecting abnormalities that can be missed in conventional imaging. Second, visualization of blood flow dynamics improves the accuracy of glaucoma diagnosis in cases with other ocular pathologies where the existing thickness map is compromised by retinal diseases such as ERM or peripapillary retinoschisis. Particularly in cases of high myopia, where RNFL defects may not be clearly observed in the red-free fundus photo, WF-OCTA can be helpful [15,43]. By providing angiographic information across a broader field than conventional imaging, WF-OCTA has the potential to enhance glaucoma diagnosis.

However, the current active clinical utilization of WF-OCTA can be limited due to some disadvantages. WF-OCTA takes a long time to acquire, requires cooperation, and can make imaging difficult for older patients, especially those with tremors. Moreover, deviation maps cannot be created, and the current embedded software only offers a combination map with RNLF, GCC, and GCIPL thickness maps since the normative database of OCTA values has not been established yet. These may make the active clinical utilization of WF-OCTA challenging.

Nevertheless, when diagnosing glaucoma becomes challenging due to other pathological changes in the eye, such as high myopia or retinal diseases, WF-OCTA can serve as a valuable adjunctive imaging technique. This study’s findings demonstrate that WF-OCTA could offer a valuable alternative to the current RNFL method for diagnosing glaucoma, especially in patients with co-existing ocular conditions. Therefore, we believe that it can be readily applied in clinical settings, particularly in cooperative patients who can endure relatively long examination times. With the assistance of FSL, the accuracy of WF-OCTA for glaucoma diagnosis could further improve, leading to significant advancements in the diagnosis and treatment of glaucoma. Moreover, FSL could be clinically applicable not only in glaucoma management but also in relatively less prevalent conditions like inherited retinal diseases or neuro-ophthalmic diseases.

This study has certain limitations. First, the advantages of FSL could have been demonstrated more accurately if we had used a wide fundus photo or OCT RNFL thickness map instead of WF-OCTA. However, this study tried to demonstrate the advantages of both FSL (new DL algorithm) and WF-OCTA (newly introduced imaging method). Second, 100 OCT RNFL thickness map images were used for feature extraction, which is still too small for pre-training. Third, it would have been more intriguing if we could compare the accuracy between diagnosis by the physician and the FSL. Fourth, OCT RNFL thickness maps were used for the training set, which are very similar to the images used in the support set (WF-OCTA). Our future studies plan to address the accuracy when using images with lower similarity as the training set.

## 5. Conclusions

This study demonstrated the effectiveness of applying DL to WF-OCTA for glaucoma diagnosis, highlighting the potential of WF-OCTA images in glaucoma diagnostics. Additionally, the application of FSL was shown to overcome the limitation of small dataset size. Utilizing FSL with imaging techniques characterized by limited data can be effective, and its applicability in various clinical settings is anticipated.

## Figures and Tables

**Table 1:** Comparison of accuracy and area under the receiver operating characteristic curve between few-shot learning and supervised learning.

	FSL			SL			FSL vs. SL: *p*-Value		
WF-OCTA	RNFL Combi	GCC Combi	Alone	RNFL Combi	GCC Combi	Alone	RNFL Combi	GCC Combi	Alone
Accuracy (%)	81	80	68	50	50	50	<0.05	<0.05	<0.05
AUC	0.930	0.881	0.701	0.802	0.799	0.640	<0.05	<0.05	<0.05

FSL = few-shot learning; SL = supervised learning; WF-OCTA = wide-field optical coherence tomography angiography; AUC = area under the receiver operating characteristic curve; RNFL = retinal nerve fiber layer; GCC = ganglion cell complex.

**Table 2:** Comparison of accuracy and area under the receiver operating characteristic curve between few-shot learning and conventional thickness value (*p*-values are expressed in the table below).

		FSL			Thickness Value		
		WF OCTA_ RNFL Combi	WF OCTA_ GCC Combi	WF-OCTA	RNFL	GCC	GCIPL
**AUC**		**0.930**	**0.881**	**0.701**	**0.870**	**0.863**	**0.782**
FSL	WF OCTA_RNFL Combi	NA	<0.05	<0.05	<0.05	<0.05	<0.05
	WF OCTA_GCC Combi	<0.05	NA	<0.05	<0.05	<0.05	<0.05
	WF-OCTA	<0.05	<0.05	NA	<0.05	<0.05	<0.05
Thickness Value	RNFL	<0.05	<0.05	<0.05	NA	0.83	0.49
	GCC	<0.05	<0.05	<0.05	0.83	NA	0.37
	GCIPL	<0.05	<0.05	<0.05	0.49	0.37	NA

FSL = few-shot learning; AUC = area under the receiver operating characteristic curve; WF-OCTA = wide-field optical coherence tomography angiography; RNFL = retinal nerve fiber layer; GCC = ganglion cell complex; GCIPL = ganglion cell–inner plexiform layer.

## Data Availability

Data are available upon reasonable request.

## Supplementary Materials

The following supporting information can be downloaded at https://www.mdpi.com/article/10.3390/biomedicines12040741/s1. Supplementary Material: Diagnosis of glaucoma and selection of control group. Figure S1: Training ResNet18 weight by using SS-OCT data to train ResNet18. Table S1: Demographic and clinical characteristics of eyes and patients in the training vs. test samples. It is written that there are 95 sheets in the training overall, but in the actual experiment, 20 sheets are randomly selected and used. Table S2: Comparison of shot, accuracy, and area under the receiver operating characteristic curve results as a function of number of shots.
