# Supplementary material for: Diagnosis of Glaucoma Based on Few-Shot Learning with Wide-Field Optical Coherence Tomography Angiography
Source: Biomedicines. 2024 Mar 27;12(4):741. doi: 10.3390/biomedicines12040741 (PMC11048300; doi:10.3390/biomedicines12040741)
Supplement: Supplementary file 1 [file biomedicines-12-00741-s001.zip › biomedicines-2906482-supplementary.pdf]

Supplementary Materials

Diagnosis of glaucoma and selection of the control group

The inclusion criteria were: best-corrected visual acuity of 20/40 or better, spherical equivalent refractive errors between +6.0 and -6.0 D, and cylinder correction < 3.0 D. The exclusion criteria were the following: history of ophthalmic surgery (e.g., glaucoma-filtering surgery; however, patients who underwent only cataract surgery were not excluded), any other ocular disease that could interfere with the visual function, any media opacity that would significantly interfere with the acquisition of OCT images, and inability to obtain high-quality OCT images (i.e., Image Quality scores < 50). For cases in which both eyes met all the eligibility criteria, one eye was randomly chosen for inclusion.

Patients with glaucoma were identified using several signs based on clinical exam. The first was the presence of a characteristic optic disc, defined as localized or diffuse neuroretinal rim thinning, increased cupping, or cup-to-disc ratio difference > 0.2 between the eyes on stereo disc photographs. The presence of RNFL defects on red-free fundus imaging was an alternative sign, irrespective of glaucomatous visual field defects. Diagnosis with all gold standards is based on a clinical exam.

Healthy control eyes were defined as those of patients with no history or evidence of intraocular surgery, intraocular pressure ≤ 21 mmHg with no history of increased intraocular pressure, absence of glaucomatous disc appearance, and normal ophthalmologic findings.

The visual field was evaluated as follows: it was considered reliable if the fixation losses were < 20%, false positive rate was < 15%, and false-negative rate was < 15%. The visual field was considered normal if the mean deviation (MD) and pattern standard deviation (PSD) lied within 95% confidence limits and the glaucoma hemifield test (GHT) yielded results within the normal limits. Eyes with glaucomatous visual field defects were defined as those with a cluster of three points with probabilities of < 5% on the pattern deviation map in at least one hemifield,

including at least one point with a probability of < 1% or a cluster of two points with a probability of < 1%, and GHT results beyond the 99% of age-specific normal limits or PSD beyond 95% of normal limits. Visual field defects were confirmed on two consecutive reliability tests (some of the longitudinal aspects were included).

Supplementary Figures

Figure S1. Training ResNet18 weight by using SS-OCT data to train ResNet18.

Pre-training with SS-OCT RNFL thickness map (12*9 mm) data, similar to WF-OCTA Combi, before learning with few-shot learning helps accumulate some knowledge in advance, enabling the model to perform the task effectively.

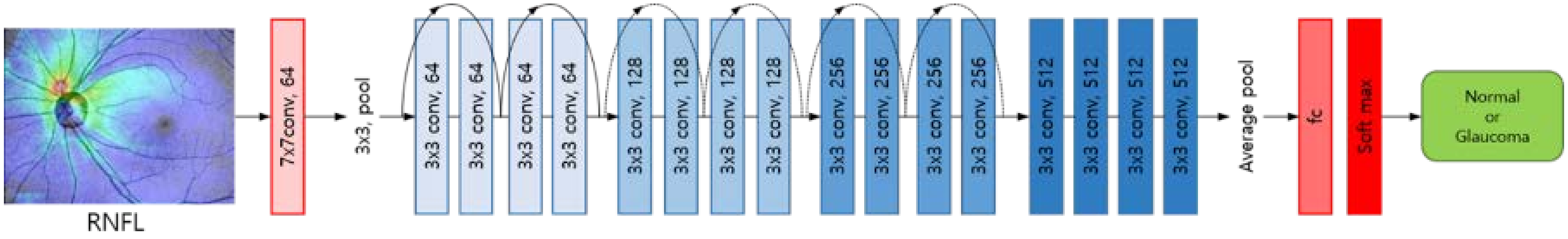

Supplementary Tables

Table S1. Demographic and Clinical Characteristics of Eyes and Patients in the Training vs Test Samples. It is written that there are 95 sheets in the training overall, but in the actual experiment, 20 sheets are randomly selected and used.

	**Overall**			**Support Set**			**Query Set**			**P**
	Overall	Normal	Glaucoma	Overall	Normal	Glaucoma	Overall	Normal	Glaucoma	
N	195	82 (42.0%)	113 (48.0%)	95	32 (33.7%)	63 (66.3%)	100	50 (50.0%)	50 (50.0%)	0.975
Sex (M)	417 (53.7%)	142 (51.8%)	275 (54.7%)	302 (55.4%)	103 (53.6%)	199 (56.4%)	115 (49.6%)	39 (47.6%)	76 (50.7%)	0.135
Age (year)	58.4 ± 15.8	53.3 ± 16.1	61.2 ± 14.9	58.2 ± 16.0	53.2 ± 16.7	60.8 ± 15.0	59.0 ± 15.3	53.6 ± 14.9	61.9 ± 14.8	0.516
SE (diopter)	−1.49 ± 2.66	−1.43 ± 2.77	−1.52 ± 2.61	−1.58 ± 2.73	−1.69 ± 2.96	−1.52 ± 2.61	−1.29 ± 2.48	−0.87 ± 2.19	−1.52 ± 2.61	0.172
AXL (mm)	25.84 ± 1.65	24.03 ± 1.72	26.13 ± 1.71	24.78 ± 1.84	24.71 ± 1.86	24.81 ± 1.83	24.54 ± 1.32	24.60 ± 1.49	24.59 ± 1.49	0.526
IOP (mmHg)	14.8 ± 2.7	15.1 ± 3.0	14.5 ± 3.0	15.0 ± 3.1	15.2 ± 3.4	15.1 ± 3.4	14.3 ± 2.5	14.9 ± 2.7	14.5 ± 3.1	0.131
MD (dB)	−6.0 ± 7.4	−2.1 ± 2.7	−7.7 ± 8.1	−5.8 ± 7.2	−2.1 ± 2.0	−7.3 ± 8.0	−6.6 ± 7.9	−2.0 ± 208	−8.6 ± 8.3	0.180
VFI (%)	84.9 ± 23.1	97.0 ± 5.0	79.9 ± 25.6	85.6 ± 22.4	97.3 ± 3.0	80.8 ± 24.9	83.3 ± 24.5	96.5 ± 7.7	77.7 ± 27.0	0.241
RNFL (μm)	84.6 ± 21.4	103.0 ± 12.0	74.6 ± 18.6	84.7 ± 21.7	103.2 ± 12.9	74.6 ± 18.6	84.5 ± 20.9	102.4 ± 9.8	74.7 ± 18.8	0.899
GCIPL (μm)	61.9 ± 9.7	69.9 ± 5.5	57.6 ± 8.7	61.9 ± 10.1	70.1 ± 5.9	57.4 ± 9.1	61.9 ± 8.7	69.3 ± 4.6	57.9 ± 7.7	0.983
GCC (μm)	94.9 ± 14.4	107.3 ± 7.4	88.1 ± 12.7	95.0 ± 15.0	108.1 ± 7.4	87.8 ± 13.2	94.6 ± 13.0	105.3 ± 7.1	88.8 ± 11.7	0.753

P = P-value for comparison between training and test sets; comparisons were performed with the chi-square test for categorical variables and

the independent t-test for continuous variables.

SE = spherical equivalent; AXL = axial length; IOP = intraocular pressure; MD = mean deviation; VFI = visual field index; RNFL = retinal nerve fiber layer; GCC = ganglion cell complex; GCIPL = ganglion cell – inner plexiform layer

Table S2. Comparison of shot, Accuracy and area under the receiver operating characteristic curve results as a function of number of shots

Data Type	2-way Accuracy (%)				AUC			
	1-shot	2-shot	5-shot	10-shot	1-shot	2-shot	5-shot	10-shot
WF-OCTA_RNFL Combi	70	74	81	81	0.747	0.883	0.889	0.930
WF-OCTA_GCC Combi	64	69	71	80	0778	0.814	0.750	0.881
WF-OCTA	49	63	54	68	0.496	0.667	0.562	0.701

AUC = area under the receiver operating characteristic curve; WF-OCTA = wide-field optical coherence tomography angiography; RNFL = retinal nerve fiber layer; GCC = ganglion cell complex
